# β-catenin has potential effects on the expression, subcellular localization, and release of high mobility group box 1 during bovine herpesvirus 1 productive infection in MDBK cell culture

**DOI:** 10.1080/21505594.2021.1926409

**Published:** 2021-05-19

**Authors:** Wenqing Fan, Weifeng Yuan, Xiuyan Ding, Liqian Zhu

**Affiliations:** a College of Veterinary Medicine, Yangzhou University and Jiangsu Co-Innovation Center for Prevention and Control of Important Animal Infectious Diseases and Zoonoses, Yangzhou China; b College of Life Sciences, Hebei University, Baoding China; c Institute of Animal Sciences, Chinese Academy of Agricultural Sciences, Beijing China

**Keywords:** BoHV-1, HMGB1, β-catenin, mitochondria

## Abstract

High mobility group box 1 (HMGB1), a ubiquitous DNA-binding protein, can be released into extracellular space and function as a strong proinflammatory cytokine, which plays critical roles in the pathogenesis of various inflammatory diseases. Here, we showed that BoHV-1 productive infection in MDBK cells at later stage significantly increases HMGB1 mRNA expression and the protein release, but decreases the steady-state protein levels. Virus infection increases accumulation of HMGB1 protein in both nucleus and mitochondria, and relocalizes nuclear HMGB1 to assemble in highlighted foci via a confocal microscope assay. Interestingly, β-catenin-specific inhibitor iCRT14 is able to increase *HMGB1* transcription and the protein release, and subcellular translocation in virus-infected cells. HMGB1-specific inhibitor, glycyrrhizin, could differentially affect virus gene transcription such as, the viral regulatory protein bICP0, bICP4 and bICP22, as well as glycoprotein gD. In summary, our data provides a novel mechanism that β-catenin signaling may regulate inflammatory response via affecting HMGB1 signaling.

## Introduction

Bovine herpesvirus 1 (BoHV-1), an enveloped DNA virus, belongs to the family *Herpesviridae* and the subfamily *Alphaherpesvirinae* [1,2]. As one of the most important pathogens in cattle, the virus infection causes a range of inflammatory diseases in distinct tissues, including the upper respiratory tract, nasal cavity, and ocular cavity, and leads to erosions in mucosal surface [3]. BoHV-1 infection has been established as a critical co-factor for development of bovine respiratory disease complex (BRDC), a known life-threatening pneumonia in cattle of all ages and breeds [4]. Generally, the erosion of mucus and immune suppression induced by virus infection may result in secondary infection by other pathogens, such as bovine viral diarrhea viruses (BVDV), bovine respiratory syncytial virus (BRSV), parainfluenza-3 virus (PI3V) and bovine coronaviruses, and bacteria including *Mannheimia haemolytica and Pasteurella multocida, Histophilus somni* and *Mycoplasma* spp, which consequently leads to BRDC development [1,2,5]. BoHV-1 infection and BRDC inflict great economic loss to the cattle industry, worldwide, which cost the US cattle industry approximately 3 billion dollars annually [6].

High mobility group box 1 (HMGB1) is a highly abundant, multifunctional, DNA-binding protein which predominantly resides in nucleus involving in regulation of gene transcription, DNA damage repair, as well as chromatin stability in physical condition [7]. It can be either passively released into the extracellular milieu by necrotic or damaged cells or secreted by activated immune cells, where it functions as a pro-inflammatory cytokine, and contributes to the pathogenesis of diverse inflammatory disease [8]. For example, the secreted HMGB1 has capacity to activate various cells, such as macrophages/monocytes, dendritic cells (DCs), and endothelial cells to promote production of proinflammatory cytokines, including tumor necrosis factor alpha (TNF-α), interleukin (IL)-6 and IL-8 [9–12]. In addition, HMGB1 has chemoattractant activity to recruit various cells such as fibroblast cells, and monocytes/monomyeloid precursors, in vitro [13,14]. Extracellular HMGB1 contributes to a variety of inflammatory diseases, such as rheumatoid arthritis, and lethal endotoxaemia and sepsis [7,11,12], which can be ameliorated by HMGB1 inhibitors, such as glycyrrhizin, via blocking HMGB1 release [9]. Accumulating studies have suggested that as a proinflammatory cytokine HMGB1 contributes to the pathogenesis of various viruses, such as respiratory syncytial virus [15–17], dengue virus [18], and hepatitis C virus [19]. However, whether BoHV-1 interacts with HMGB1 is currently unknown. By virtue of its critical roles in orchestrating inflammatory responses, studying the interplay between HMGB1 and BoHV-1 infection is important to understand the virus pathology.

The canonical Wnt/β-catenin signaling pathway is required for the regulation of cell proliferation, cell survival, development and tissue regeneration [20,21]. It can also regulate the production of multiple inflammatory cytokines. Vice versa, it can be activated by a variety of inflammatory cytokines [22,23]. We have recently reported that β-catenin signaling is additionally activated to support BoHV-1 latent infection in sensory neurons, and productive infection in cell culture [24–28]. Whether β-catenin signaling has effects on HMGB1 pathway in virus-infected cells remains poorly understood.

In quiescent cells, cytosol β-catenin is constitutively associated with adenoma polyposis coli (APC), axin, glycogen synthase kinase 3β (GSK-3β), and casein kinase I [29], assembling in β-catenin destruction complex, which leads to polyubiquitination and consequent degradation of β-catenin by the proteasome [21]. Once activated, β-catenin destruction complex is disassociated, β-catenin is consequently stabilized and accumulates in nucleus, where it interacts with members of the T-cell factor (TCF) family of DNA-binding proteins specifically bound to the consensus site 5′(A/T)(A/T)CAAAG3′ [30,31]. The binding of β-catenin to TCF family members displaces the bound corepressors and recruits coactivators such as cyclic AMP-response element-binding protein (CREB)-binding protein (CBP) and its close relative p300 to the carboxy-terminal transactivation domain of β-catenin to activate target genes [21,32]. It has been reported that the promoter sequence of HMGB1 contains two putative TCF binding elements which can be bound by β-catenin in rKyse150 cells [33]. Intriguingly, another independent studies show that β-catenin signaling is activated after overexpression of HMGB1 in H1299 cells, but inactivated by knockdown of HMGB1 in A549 cells [34]. Hereby, both β-catenin and HMGB1 may exert reciprocal positive effects on the expression of each other, providing a clue that BoHV-1 infection may have effects on HMGB1 signaling. Here, we hypothesized that BoHV-1 infection has an influence on HMGB1 signaling transduction, which may be achieved via β-catenin pathway.

In this study, we demonstrate that HMGB1 facilitates BoHV-1 productive infection in MDBK cells, and virus infection at later stage significantly increases HMGB1 release and alters HMGB1 subcellular localization. Importantly, β-catenin is potentially involved in the regulation of HMGB1 signaling pathway in virus-infected cells.

## Material and methods

### Cells and viruses

Madin-Darby bovine kidney (MDBK) cells (purchased from Chinese model culture preservation center, Shanghai, China) were cultured in DMEM containing 10% fetal bovine serum (FBS). BoHV-1 (NJ-16-1) isolated from commercial bovine semen [35] was propagated in MDBK cells. Aliquots of virus stocks were titered in MDBK cells and stored at −80°C.

### Antibodies and chemical reagents

iCRT14 (MedChemExpress, cat# HY16665), Glycyrrhizin acid (MedChemExpress, cat# HY-N0184). HMGB1 rabbit monoclonal antibody (mAb) (ABclonal, cat# A19529), RAGE rabbit polyclonal antibody (pAb) (ABclonal, cat# A1395), β-catenin rabbit mAb (Abcam, cat# ab32572), laminA/C mouse mAb (Santa Cruz Biotechnology, cat# sc-376,248), Cox IV rabbit pAb (Cell Signaling Technology, cat# 4844), β-Tubulin rabbit pAb (Abclonal, cat# AC015), GAPDH mouse mAb (Cell Signaling Technology, cat# 2118), BoHV-1 gC mouse mAb (VMDR Inc, cat# F2), BoHV-1 gD mouse mAb (VMDR Inc, cat# 1B8-F11), Alexa Fluor 488®-conjugated goat anti-rabbit IgG (H + L) (Invitrogen, cat# A-11,008), HRP- (horseradish peroxidase-) conjugated goat anti-mouse IgG (Cell Signaling Technology, cat# 7076), and goat anti-rabbit IgG (Cell Signaling Technology, cat# 7074).

### Western blot analysis

MDBK cells were seeded into 60 mm dishes and cultured overnight. The cells were infected with BoHV-1 (MOI = 0.1) or UV-inactivated virus (MOI = 0.1) for 1 hour (h), after washing three time with PBS fresh medium were replaced and incubated for further infection. At 24 hours post infection (hpi), cell lysates were prepared using lysis buffer (1% Triton X-100, 50 mM sodium chloride, 1 mM EDTA, 1 mM EGTA, 20 mM sodium fluoride, 20 mM sodium pyrophosphate, 1 mM phenylmethylsulfonyl fluoride, 0.5 g/mL leupeptin, 1 mM benzamidine, and 1 mM sodium orthovanadate in 20 mM Tris-HCl, pH 8.0).

To determine the effects of the detected chemical inhibitors, cell cultures were treated with either DMSO vehicle or inhibitors such as iCRT14 or Glycyrrhizin acid at indicated concentration for 1 h at 37°C in a humidified incubator with 5% CO_2_. Cells were infected with BoHV-1 (MOI = 0.1) for 1 h in the presence of the chemicals indicated. After washing three times with PBS, fresh medium containing either DMSO or inhibitor was replaced. At 24 hpi, the cell lysates were prepared and centrifugated at 13,000 rpm for 10 min at 4°C. The clarified supernatants were collected and boiled together with Laemmli sample buffer for 10 min; samples were subsequently separated by 8% or 10% SDS-PAGE and proteins were transferred onto PVDF membranes (Bio-Rad, cat# 1,620,177). After blocking with 5% nonfat milk in PBS for 1 h at room temperature, the membranes were incubated with primary antibodies diluted in 5% bovine serum albumin in PBS, overnight at 4°C. After extensive washing with PBST (0.1% Tween-20 in PBS), membranes were incubated with secondary antibodies of either anti-rabbit or anti-mouse for 1 h at room temperature. After extensive washing with PBST, protein bands were developed onto film by using Clarity Western ECL substrate (Bio-Rad, cat# 1,705,061).

### Immunofluorescence assay (IFA)

MDBK cells seeded into 8-well chamber slides (Nunc Inc., IL, USA) were mock infected or infected with BoHV-1 (MOI = 0.1) for 24 h. Cells were fixed with 4% paraformaldehyde in PBS for 10 min at room temperature, permeabilized with 0.25% Triton X-100 in PBS for 10 min at room temperature, and blocked with 1% BSA in PBST for 1 h followed by incubation with indicated antibodies in 1% BSA in PBST overnight at 4°C. After three washings, cells were incubated with Alexa Fluor 488®-conjugated goat anti-rabbit IgG (H + L) (Invitrogen, cat# A-11,008, 1 : 1500 dilution) for 1 h in the dark at room temperature. After three washings, DAPI (4′,6-diamidino-2-phenylindole) staining was performed to visualize nuclei. Slides were covered with coverslips by using antifade mounting medium (Electron Microscopy Sciences, cat# 50–247-04). Images were captured using a confocal microscope (Leica).

### Relative quantification of mRNA by qRT-PCR

MDBK cells of 5 × 10^5^ were seeded into 6-well plates and cultured overnight. The cells were pretreated with either DMSO vehicle or iCRT14 (10 μM) or glycyrrhizin acid (200 μM) in 1.5 ml DMEM. After treatment for 1 h, the cells were infected with BoHV-1 with MOI of 0.1 in 1 ml of DMEM medium containing indicated chemicals. After infection for 1 h at 37°C in the CO_2_ incubator, the inoculation was removed, the cells were washed three time by using PBS, then fresh medium of 1.5 ml containing 2% FBS together with indicated chemicals was replaced. At 24 hpi, the RNA was purified using a TRIzol LS reagent (Ambion, cat# 10,296,010) following the manufacturer’s instructions. Freshly prepared total RNA was used for real-time quantitative PCR to measure mRNA levels of HMGB1, bICP4, bICP0, bICP22, and gD, as well as 18s RNA with specific primers as previously described in the references [36–40]. Analysis of 18s RNA was used as an internal control. Real-time PCR was carried out using the ABI 7500 fast real-time system (Applied Biosystems, CA). The expression levels of the tested genes were normalized to that of the 18s RNA gene. The relative mRNA level of each gene was calculated using the method (2^−*ΔΔ*CT^) by a comparison to the control.

### siRNA knockdown

siRNA targeting HMGB1 (GCAUUCUUUGUGCAAACUUTTAAGUUUG CACAAAGAAUGCTT) were purchased from Genepharma (Shanghai, China). The scrambled siRNA was also provided by Genepharma (Shanghai, China). siRNA transfection was performed with transfection reagent siRNA-Mate (Genepharma) according to the manufacturer’s specifications. Efficiency of these siRNA was characterized by qRT-PCR.

### Detection of released HMGB1 with ELISA

To determine the effects of BoHV-1 infection had on HMGB1 release, MDBK cells in 6-wells plates were mock infected or infected with either BoHV-1 or UV-inactivated virus at an MOI of 0.1. After infection for 24 h, the supernatants were collected, and clarified by brief centrifugation at 12000 rpm for 5 min. To validate the effects, the cells were treated with glycyrrhizin acid at a concentration of 100 μM for 24 h as a control. HMGB1 protein levels in the supernatants were detected with commercial ELISA kits (Solarbio, cat# SEKH-0409).

To investigate the effects of iCRT14 had on HMGB1 release in a context with or without BoHV-1 infection, the cells were treated with either DMSO or iCRT14 (10 μM) during virus infection plus a pretreatment for 1 h. To validate the effects, the cells of either with or without infection were treated with glycyrrhizin acid. After infection for 24 h, the supernatants were clarified and subjected to the detection of HMGB1 protein concentration with commercial ELISA kits (Solarbio, cat# SEKH-0409), respectively.

## Results

### BoHV-1 infection increases HMGB1 mRNA expression and protein release

**Figure 1.**
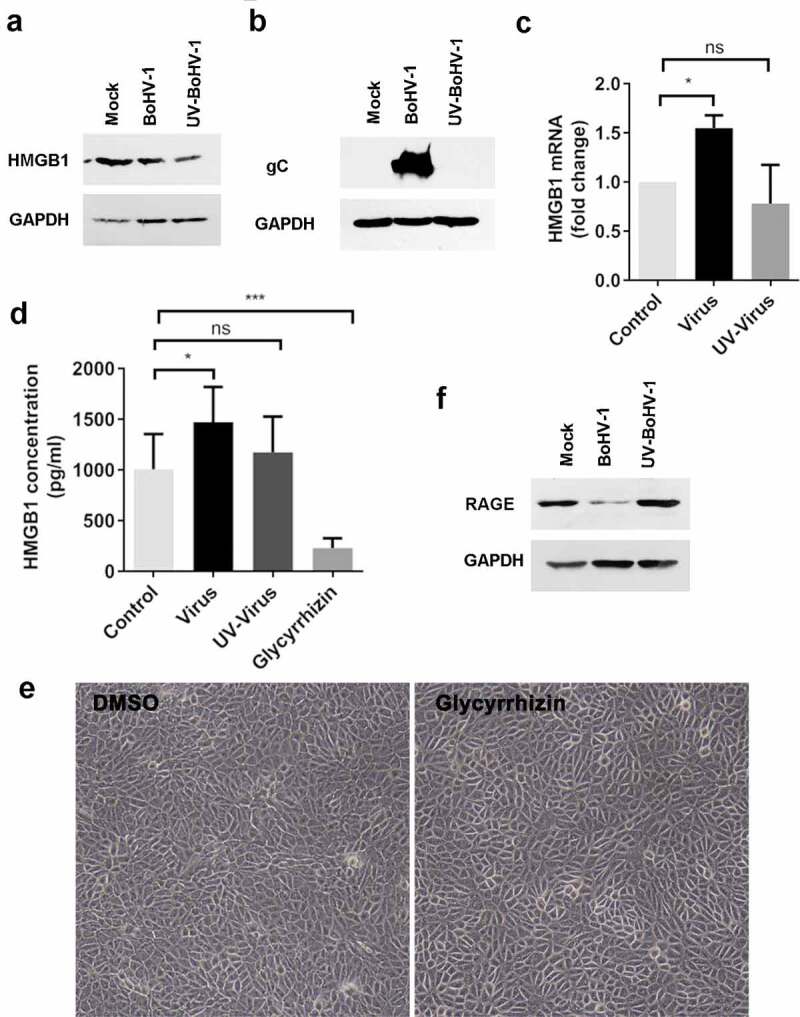
Effects of BoHV-1 infection has on HMGB1 expression and release. (A, B and F) MDBK cells in 60 mm dishes were mock infected or infected with either BoHV-1 or UV-inactivated virus at an MOI of 0.1 for 24 h. The cell lysates were then prepared to detect the expression of HMGB1(a), viral envelope protein gC (b), and RAGE (f), by Western blot using indicated antibodies. (c) MDBK cells in 6-well plates were mock infected, or infected with either BoHV-1 or UV-inactivated virus at an MOI of 0.1 for 24 h. At 24 hpi, the total RNA was purified, and the mRNA levels of HMGB1 were detected with qRT-PCR. (d) MDBK cells in 6-well plates were mock infected, or infected with either BoHV-1 or UV-inactivated virus at an MOI of 0.1 for 24 h. In parallel, the cells treated with 200 μM of glycyrrhizin acid were used as controls. After infection for 24 h, the supernatants were collected, and clarified by centrifugation. The protein concentrations of HMGB1 were detected by using a commercial ELISA kit. (e) MDBK cells in 24-well plates were mock treated with either DMSO control or glycyrrhizin acid (200 μM). At 24 h post treatment, the cell morphology was observed under a light microscope. Images shown are representative of two independent experiments (magnification: 200×). Significance was assessed with student *t*-test (* *p* < 0.05, *** *p* < 0.001); ns: not significant

To understand whether HMGB1 is involved in BoHV-1 productive infection, we initially detected HMGB1 protein levels in MDBK cells (a widely used bovine kidney cell line that support virus replication) following virus infection at later stages [at 24 hours post infection (hpi).] Relative to that in the mock-infected cells, steady-state HMGB1 protein levels were decreased following virus infection (Figure 1a). UV-inactivated virus could bind to the virus receptors and enter the cells, but could not express viral genes [41]. Here, complete inactivation of the virus is confirmed by detection of viral glycoprotein gC in virus infected cells but not in the cells exposed to UV-inactivated virus (Figure 1b). Surprisingly, UV-inactivated viral particles also were able to induce HMGB1 protein depletion (Figure 1a), suggesting that the depletion of HMGB1 is not completely dependent on intact viral replication cycles. Relative to the uninfected cells, the mRNA expression levels of HMGB1 are increased to approximately 1.55-fold following virus infection, but not affected by the UV-inactivated virus (Figure 1c). Obviously, the decreased steady-state protein levels of HMGB1 did not corroborate the increased mRNA levels in response to virus infection.

Since HMGB1 protein can be released into extracellular milieu, the released HMGB1 protein levels induced by virus-infected cells were examined with commercial ELISA kit. As a result, virus infection significantly promoted HMGB1 release in MDBK cells, compared to that of uninfected controls (Figure 1d). UV-inactivated virus had no effects on HMGB1 release (Figure 1d). The concentrations of HMGB1 protein for the results shown in Figure 1d were: uninfected controls (1005.67 ± 340.00 pg/mL), virus infection (1471.50 ± 345.28 pg/mL), infection by UV-inactivated virus (1174.00 ± 352.43 pg/mL), and treatment by 200 μM of glycyrrhizin (227 ± 99.60 pg/mL) (Figure 1d). Glycyrrhizin is known to inhibit HMGB1 release [42]. At a concentration of 200 uM, it did not show evident cytotoxicity to MDBK cells in comparison to DMSO controls, as judged by either cell morphology (Figure 1e) or by Trypan-blue exclusion test [43] (data not shown), but it significantly decreased HMGB1 release in MDBK cells, suggesting that the decreased release of HMGB1 by glycyrrhizin is not caused by the cytotoxicity to the cell culture. Thus as a control it validates the findings that virus infection promotes HMGB1 release. Taken these data together, BoHV-1 infection promotes HMGB1 release, which may consequently account for the depletion of cellular HMGB1.

It is known that extracellular HMGB1 protein could bind to its cognate receptor, advanced glycation end products (RAGE), thereby stimulates a series of cellular signal pathways. We then examined the protein expression of RAGE following virus infection. Relative to the mock-infected controls, the steady-state protein levels of RAGE were significantly decreased in the cells infected by virus but not by UV-inactivated virus (figure 1f). The depletion of RAGE protein following virus infection may have influence on the signaling transduction stimulated by released HMGB1.

### BoHV-1 infection relocalizes nuclear HMGB1 and promotes accumulation of HMGB1 protein in both nucleus and mitochondria

**Figure 2.**
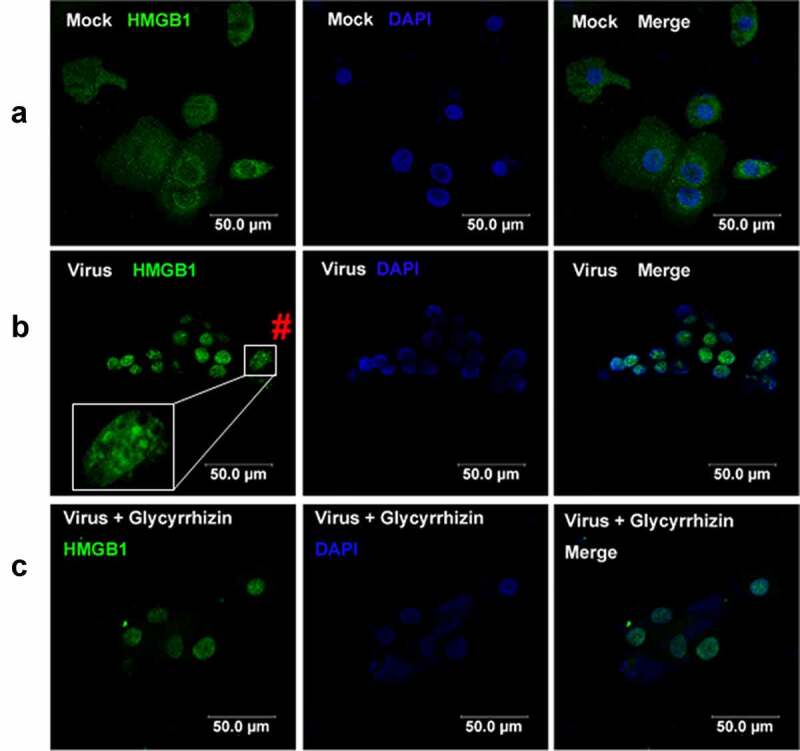
BoHV-1 infection altered the subcellular localization of HMGB1 protein. MDBK cells in 2-well chamber slides were mock infected (a) or infected with BoHV-1 (MOI = 0.1) (b) for 24 h. After three washings with PBS, cells were fixed with 4% formaldehyde, and HMGB1 protein was detected by IFA. DAPI staining was used to stain nuclear DNA. # denoted zoom-in cells showing typical staining of HMGB1 foci. (c) The cells treated with glycyrrhizin acid (200 μM) throughout infection plus a pretreatment were used as controls because glycyrrhizin acid is known to be able to block HMGB1 release and the nucleocytoplasmic translocation. Images were obtained by performing confocal microscopy (Leica)

To understand whether BoHV-1 infection has influences on HMGB1 subcellular localization, an immunofluorescence assay (IFA) was performed at 24 hpi. In the mock-infected MDBK cells, HMGB1 locates throughout the cells, and highlighted staining is readily observed around the rim of nucleus (Figure 2a). In addition, HMGB1 in the cytoplasmic compartments showed characteristic granular staining patterns (Figure 2a). After virus infection, staining of HMGB1 is predominantly observed in the nucleus, and highlighted staining around the rim of nucleus as observed in uninfected cells was not readily detected (Figure 2b). Of note, to readily detect HMGB1 protein in uninfected cells and in virus-infected cells, distinct exposure time was required, and thus one cannot compare HMGB1 protein levels to that in Figure 1a.

Though it was dispersed throughout the nucleus, highlighted staining of HMGB1 foci was readily observed in nucleus in virus-infected cells (Figure 2b). The zoom-in cells as denoted by symbol #, showed typical HMGB1 foci (Figure 2b). While the highlighted HMGB1 foci were not readily detected in uninfected cells (Figure 2a). When the virus-infected cells were treated by glycyrrhizin, HMGB1 protein mainly located at the nucleus with HMGB1 foci turning to blurry (Figure 2c). As a control, these effects of glycyrrhizin had on HMGB1 mobilization validated our findings that BoHV-1 infection leads to subcellular relocalization of HMGB1.

**Figure 3.**
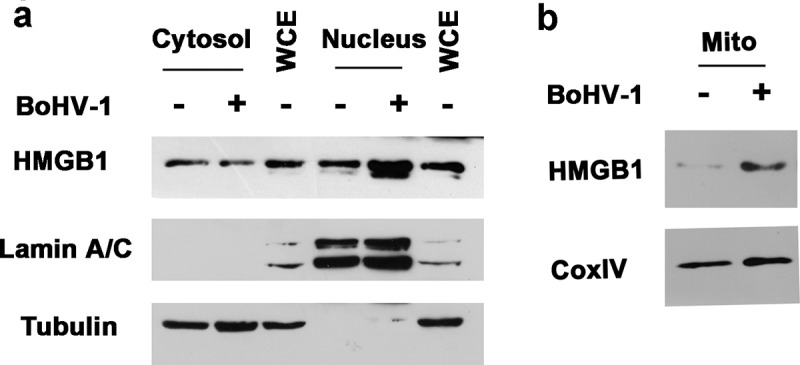
Effects of BoHV-1 infection on HMGB1 localization in nucleus and mitochondria. MDBK cells in 60 mm dishes were mock infected or infected with BoHV-1 (MOI = 0.1) for 24 h. The cells were collected for isolation of fractions of nucleus, cytoplasm (containing mitochondria), and mitochondria by using two commercial cellular fraction purification kits (Beyotime Biotechnology, cat# P0027, and cat# C3601). (a) HMGB1 protein levels in cytosol and nuclear fractions, as well as whole cell extracts (WCE) used as controls, were detected by Western blot. Tubulin and LaminA/C were detected and used as a protein loading control for cytoplasm and nucleus protein, respectively. LaminA/C in the cytosol fractions and Tubulin in the nucleus fractions were detected by Western blot to determine if these fractions were contaminated by the counterpart fractions. (b) HMGB1 protein levels in the mitochondria fractions were detected by Western blot. Cox IV was detected and used as protein loading control. Data shown are representative of three independent experiments

Of note, accumulating studies have suggested that HMGB1 mainly resides in the nucleus in quiescent cells, which is largely different from what we observed in Figure 2a. Thus, another independent method was employed to further analyze HMGB1 localization in MDBK cells, in which cellular fractions of both cytoplam and nucleus were purified by using a commercial nucleus isolation kit, and subjected to Western blotting. In line with what we observed in Figure 2a, HMGB1 protein can be clearly detected in the cytoplasmic fractions by Western blot (Figure 3a). In addition, we found that relative to the mock-infected controls, HMGB1 protein levels were increased in the nucleus fractions, but decreased in the cytoplasmic fractions following virus infection (Figure 3a). LaminA/C, a marker for nuclear protein was not detected in the cytoplasmic fraction, and β-tubulin, a marker for cytoplasmic protein was not detected in the nuclear fractions, suggesting that neither fractions are contaminated by the counterpart (Figure 3a), which validates the findings that part of HMGB1 protein locates at cytoplasm in uninfected cells, and virus infection promotes accumulation of HMGB1 protein in the nucleus but the total protein levels in cytoplasm were decreased. Overall, these data support the findings in the IFA studies as shown in Figure 2 A and 2B.

It should be noted that the cytoplasmic fractions purified by the commercial nucleus isolation kit (Beyotime Biotechnology, cat# P0027) contain mitochondria. To detect whether HMGB1 resides in mitochondria in MDBK cells, and whether it is influenced by virus infection, the mitochondria proteins were purified by using a commercial kit (Beyotime Biotechnology, cat# C3601) specific for the isolation of mitochondria from cells. When the purified mitochondrial fractions were subjected to detection of HMGB1 protein via Western blot, we found that HMGB1 protein in mitochondria was increased by virus infection, relative to that in mock-infected controls (Figure 3b). So even though total levels of HMGB1 protein were reduced in cytoplasm, the accumulation in mitochondria is increased following virus infection. We suggested that virus infection promotes translocation of HMGB1 protein into nucleus, mitochondira, and extracellular spaces, which consequently leads to depletion of cytoplasmic HMGB1.

Taken these data together, virus infection promotes accumulation of HMGB1 protein in both nucleus and mitochondria. We speculated that cytosol is the major source of HMGB1 protein flowing into different directions driven by virus infection. And the increased accumulation of nuclear HMGB1 may come from either the nuclear membrane rim-attached HMGB1 or cytoplasm. The increased accumulation of HMGB1 in the mitochondria should come from cytoplasm.

### β-catenin-specific inhibitor iCRT14 promotes HMGB1 release

**Figure 4.**
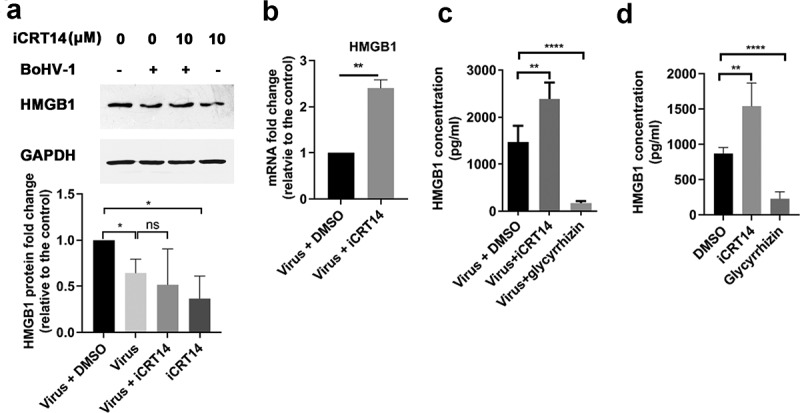
The effects of β-catenin-specific inhibitor iCRT14 on HMGB1 release. (a) MDBK cells in 60 mm dishes were pretreated with iCRT14 (10 μM) for 1 h, then they were mock infected or infected with BoHV-1 (MOI = 0.1) in the presence of iCRT14 (10 μM) or DMSO control. After infection for 24 h, cell lysates were prepared for Western blotting to detect the steady-state expression of HMGB1. The band intensity was analyzed using free software Image J. Data shown are representative of three independent experiments. (b) MDBK cells in 6-well plates pretreated with iCRT14 (10 μM) for 1 h were infected with BoHV-1 (MOI = 0.1) in the presence of iCRT14 (10 μM) or DMSO control. At 24 hpi, the RNA were extracted and mRNA levels of HMGB1 were detected by qRT-PCR. (c and d) MDBK cells in 6-well plates pretreated either with DMSO control or chemical inhibitors, including iCRT14 (10 μM) and glycyrrhizin acid (200 μM), were infected with BoHV-1 at an MOI of 0.1 for 24 h in the presence of indicated inhibitors (c). In parallel, the uninfected cells were treated with indicated inhibitors for 24 h (d). The supernatants were collected, and clarified by centrifugation. The protein concentrations of HMGB1 were detected by using a commercial ELISA kit. Data shown are means of three independent experiments. Significance was assessed with student *t*-test (** *p* < 0.01, **** *p* < 0.0001)

It has been reported that β-catenin/TCF4 complex is able to transactivate HMGB1 [44]. To understand whether this effect is still there in the context of virus infection, we detected the protein and RNA levels of HMGB1 in the presence of β-catenin-specific inhibitor iCRT14 which inhibits β-catenin dependent transcription by interfering with the interaction between β-catenin and TCF family members [45]. We found that the steady-state protein levels of HMGB1 are reduced by iCRT14 in uninfected cells but not in virus infected cells in comparison to the individual controls (Figure 4a). The mRNA expression levels of HMGB1 were increased to approximately 2.4-fold by the treatment of iCRT14 in virus-infected cells, relative to that of the DMSO controls (Figure 4b), which supports the findings that iCRT14 significantly increased the release of HMGB1 in virus-infected cells validated by glycyrrhizin known to block HMGB1 release (Figure 4c). The concentrations of HMGB1 protein released by virus-infected cells were 1471.50 ± 345.28 pg/mL, which were increased to 2387.3 ± 349.15 pg/mL by iCRT14, while they were decreased to 173.17 ± 44.09 pg/mL by glycyrrhizin (Figure 4c). Relative to the DMSO controls, iCRT14 also had strong capacity to stimulate HMGB1 release in uninfected cells (Figure 4d). In uninfected cells, the released protein levels of HMGB1 were 868 ± 86.63 pg/mL in the presence of DMSO control, which were increased to 1540 ± 326.7 pg/mL by iCRT14, and decreased to 227 ± 99.60 pg/mL by glycyrrhizin. These data suggested that iCRT14 is able to increase HMGB1 protein release either in the presence or absence of virus infection.

### β-catenin specific inhibitor iCRT14-induced cell apoptosis partially accounts for the enhanced release of HMGB1 during BoHV-1 productive infection

**Figure 5.**
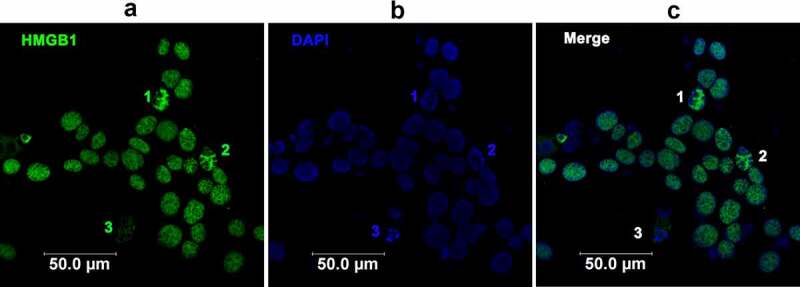
The effects of β-catenin-specific iCRT14 on cell apoptosis induced by BoHV-1 infection. MDBK cells in 2-well chamber slides pretreated with iCRT14 (10 μM) for 1 h, were infected with BoHV-1 (MOI = 0.1) for 24 h. After three washings with PBS, cells were fixed with 4% formaldehyde, and HMGB1 protein was detected by IFA. DAPI staining was used to stain nuclear DNA. Images were captured by confocal microscopy (Leica)

HMGB1 can be released from both activated and dying cells [46]. β-catenin signaling is essential for cell survival [47]. iCRT14 at a concentration of 10 μM did not show cytotoxicity to MDBK cells in the absence of virus infection as we described elsewhere [48]. Here, we found that apoptotic bodies are readily detected in virus-infected cells in the presence of iCRT14 (Figure 5, numbered 1 to 3), suggesting that apoptosis is induced in virus-infected cells by iCRT14. Of note, the cells were infected at low MOI of 0.1, where cell apoptosis were not readily detected at 24 hpi in the presence of DMSO (Figure 2b). It is highly possible that iCRT14 promotes HMGB1 protein release partially via induction of cell death. Moreover, the highlighted staining of HMGB1 foci induced by virus infection (Figure 2b) were not readily observed after iCRT14 treatment (Figure 5). While pronounced staining of HMGB1 was mainly located at the space between apoptotic bodies (Figure 5, numbered 1–3), indicating that inhibition of β-catenin signaling by iCRT14 leads to relocalization of nuclear HMGB1 protein. Taken together, iCRT14 treatment promotes cell apoptosis and HMGB1 relocalization in virus-infected cells may partially enhance HMGB1 release. Considering that distinct exposure times are required for the capture of nuclear HMGB1 either with or without iCRT14 treatment, one cannot compare either cytosol or nuclear HMGB1 levels between Figure 2C and 5.

### β-catenin is potentially involved in subcellular relocalization of HMGB1 during BoHV-1 productive infection

**Figure 6.**
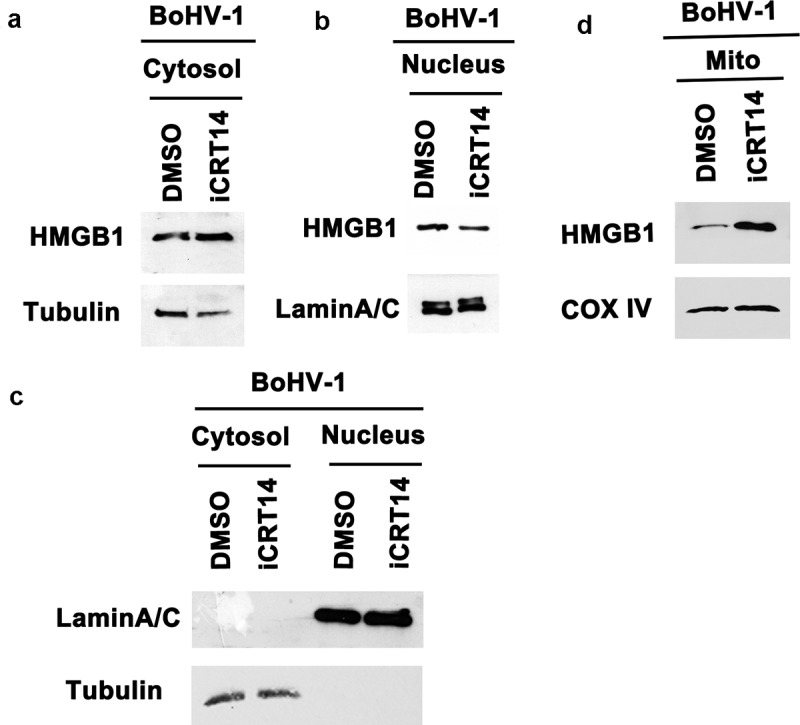
Effects of β-catenin-specific inhibitor iCRT14 on HMGB1 translocation. MDBK cells in 60 mm dishes pretreated with either DMSO control or iCRT14 (10 μM) for 1 h, were infected with BoHV-1 (MOI = 0.1) for24 h in the presence of indicated chemicals, respectively. The cells were collected for isolation of fractions of nucleus, cytoplasm, and mitochondria by using two commercial cellular fraction purification kits (Beyotime Biotechnology, cat# P0027, and cat# C3601). (A, B and D) HMGB1 protein levels in the fractions of cytosol (a), nucleus (b), and mitochondria (d) were detected by Western blot. Tubulin, LaminA/C, and Cox IV were detected and used as protein loading control of cytoplasm, nucleus, and mitochondria, respectively. (c) LaminA/C in the cytosol fractions and Tubulin in the nuclear fractions were detected by Western blot to determine if these fractions were contaminated by the counterpart fractions. Data shown are representative of three independent experiments

To further understand the mechanism of how HMGB1 mobilization is affected by iCRT14 in virus infected cells, cellular fractions of cytosol, nucleus, and mitochondria were isolated by using commercial kit. As a result, HMGB1 protein levels were significantly increased in cytoplasm, but decreased in nucleus following iCRT14 treatment, relative to that in mock-treated controls (Figure 6a and 6b). LaminA/C in the cytosol fraction and tubulin in the nucleus fraction were rarely detected, indicating that these cellular fractions were not contaminated (Figure 6c), which validated the translocation of HMGB1 in virus-infected cells induced by iCRT14. Since the cytoplasm fractions purified by using nucleus isolation kit (Beyotime Biotechnology, cat# P0027) contain mitochondria, and virus infection promotes accumulation of HMGB1 in mitochondria (Figure 3b), we wondered whether the translocation to mitochondria is affected by iCRT14. So mitochondria are isolated by using a kit specific for the purification of mitochondria. As a result, the accumulation of HMGB1 protein in virus-infected mitochondria was further increased in response to iCRT14 treatment, relative to that in mock-treated infected controls (Figure 6d). These findings suggest that in BoHV-1-infected cells inhibition of β-catenin signaling by iCRT14 promotes translocation of HMGB1 into mitochondria, but decreased into the nucleus. So β-catenin signaling is potentially involved in HMGB1 subcellular translocation in virus-infected cells.

### HMGB1 plays important roles in BoHV-1 productive infection

**Figure 7.**
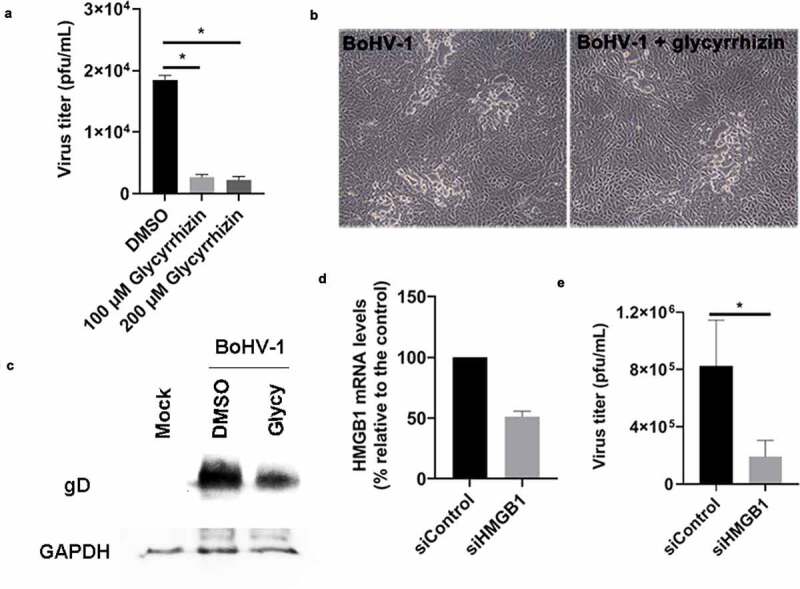
The effects of HMGB1 inhibitor glycyrrhizin on BoHV-1 productive infection. (a and b) MDBK cells in 24-well plates pretreated with either DMSO control or glycyrrhizin acid (100 and 200 μM) were infected with BoHV-1 (MOI = 0.1) for 24 h along with treatment of indicated compounds. The cell cultures were collected and virus titers were determined in MDBK cells (a). The cell morphology was observed under a light microscope. Images shown are representative of three independent experiments (magnification: 200×) (b). (c) MDBK cells in 60 mm dishes pretreated with either DMSO control or glycyrrhizin acid (200 μM) were infected with BoHV-1 (MOI = 0.1) for 24 h. Throughout infection the cells were treated with either DMSO control or glycyrrhizin acid. The cell lysates were prepared and subjected to Western blot to detect virus glycoprotein gD. (d) MDBK cells (60–70% confluent) were transfected with either scrambled siRNA (siControl) or HMGB1 specific siRNA (siHMGB1) (200 pmol). At 48 hours after transfection, total RNAs were prepared and analyzed by qRT-PCR to measure HMGB1 mRNA levels. HMGB1 mRNA in MDBK cells transfected with siHMGB1 was normalized to that transfected with siControl, which was set at 100%. (e) MDBK cells (60–70% confluent) were transfected with either siControl or siHMGB1 (200 pmol). At 48 hours after transfection, cultures were infected with BoHV-1 using an MOI of 1. Twenty-four h after infection virus yield was examined with results expressed as pfu/ml. Data shown are represent of three independent experiments. Results are the mean of three independent experiments, with error bars showing standard deviations. Significance was assessed with student *t*-test (* *p< 0.05)*

Glycyrrhizin could bind to HMGB1 protein and blocks the nucleocytoplasmic translocation, thereby inhibiting its extracellular release [49]. We found that glycyrrhizin relocalizes nuclear HMGB1 (Figure 2c). In this study, the effects of HMGB1 had on BoHV-1 productive infection were analyzed via treatment of cells with glycyrrhizin during virus infection. As a result, glycyrrhizin (100 or 200 uM) significantly decreased virus titers compared to the DMSO controls (Figure 7a). The virus titers for the results shown in Figure 7a were DMSO controls (1.85 × 10^4^ pfu/mL), 100 uM glycyrrhizin (2.67 × 10^3^ pfu/mL), and 200 uM glycyrrhizin (2.20 × 10^3^ pfu/mL). Of note, glycyrrhizin at a concentration of 200 uM did not show evident cytotoxicity to MDBK cells when the incubation period was for 24 h in a context without virus infection (Figure 1e). When cell morphology was examined, 200 µM glycyrrhizin moderately alleviates virus infection-induced cytopathology effects in MDBK cells (Figure 7b), suggesting that the reduced virus yield by glycyrrhizin is not due to the cytotoxicity to MDBK cells. The content of viral glycoprotein gD in virus infected-cells was obviously reduced by glycyrrhizin, relative to that of DMSO controls (Figure 7c), which supported the findings that glycyrrhizin inhibits virus replication.

Since the chemical inhibitor glycyrrhizin may have an off-target effect, we decided to use specific siRNA-mediated knockdown to verify the specific role of HMGB1 in BoHV-1 replication in MDBK cells. The siRNA can efficiently knock down HMGB1 expression in MDBK cells as determined by qRT-PCR (Figure 7d). When compared to the scrambled control siRNA (denoted as siControl), the HMGB1 RNA levels were reduced by approximately 50% by HMGB1 specific siRNA (denoted as siHMGB1) (Figure 7d). We then transfected MDBK cells with either the control siRNA, or the siHMGB1, and at 48 h post transfection, we infected cells with BoHV-1. As a result, siHMGB1 reduced virus production approximately 4.3-fold relative to siControl (Figure 7e). The virus titers shown in Figure 7e were scrambled siRNA (8.26 × 10^5^ pfu/mL), and siHMGB1 (1.91 × 10^5^ pfu/mL). Therefore, using both chemical inhibitor and siRNA-specific knockdown, we have shown that HMGB1 plays an important role in BoHV-1 productive infection in MDBK cells.

**Figure 8.**
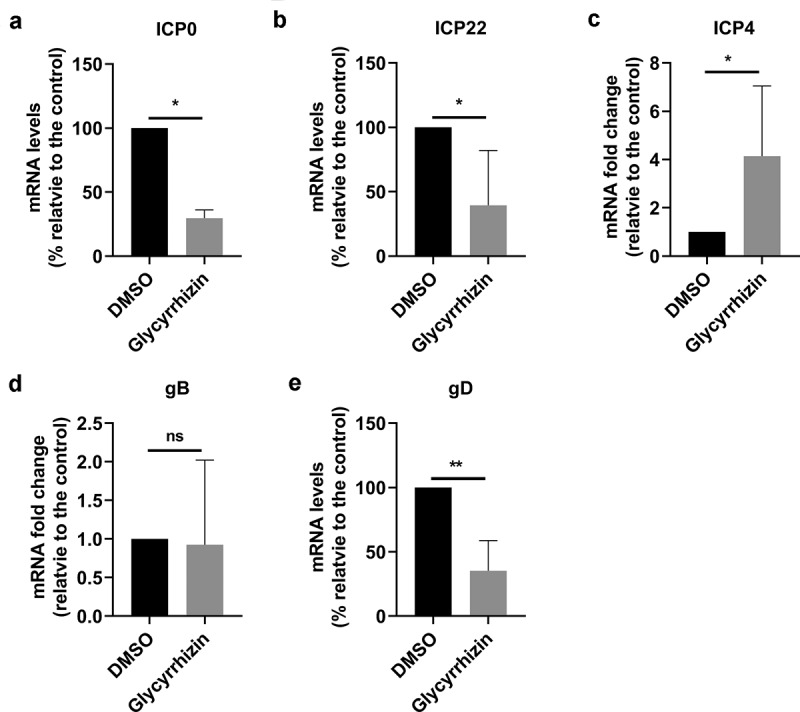
The effects of HMGB1 inhibitor glycyrrhizin on BoHV-1 mRNA expression during productive infection. MDBK cells in 6-well plates pretreated with glycyrrhizin acid (200 u M) for 1 h were infected with BoHV-1 (MOI = 0.1) in the presence of glycyrrhizin acid or DMSO control throughout infection. At 24 hpi, the RNA were extracted and mRNA levels of virus encoded protein ICP0 (a), ICP22 (b), ICP4 (c), gB (d) and gD (e) were detected by qRT-PCR, which were normalized to that of the 18sRNA. Results are the mean of three independent experiments, with error bars showing standard deviations. Significance was assessed with student *t*-test (* *p*< 0.05, ns, not significant)

Nuclear HMGB1 interacts with nucleosomes, transcription factors, and histones to regulate cellular gene transcription [50]. Thus, we detected whether HMGB1 had effects on viral mRNA expression by using qRT-PCR. The mRNA levels of viral regulatory proteins bICP0 and bICP22 were significantly reduced by glycyrrhizin, which are reduced to approximately 30% and 39.5% relative to that of mock-treated by DMSO (Figure 8a and 8b). In contrast, the mRNA levels of bICP4, a viral regulatory protein, were increased nearly 4-fold by glycyrrhizin in comparison to that of mock treatment by DMSO (Figure 8c). The mRNA expression of the envelope glycoprotein gB was not affected by glycyrrhizin (Figure 8d). In consistent with the decreased protein expression of gD in Western blot (Figure 7c), glycyrrhizin significantly inhibits the mRNA expression of gD (Figure 8e). Take these data together, HMGB1 has differential effects on BoHV-1 gene expression.

## Discussion

Accumulating studies indicated that HMGB1 regulates viral replication cycles with different mechanisms for distinct viruses. For instance, intracellular HMGB1 binds to HCV genomic RNA at 5ʹuntranslated region (UTR) to promote HCV replication [19]. HMGB1 binds to oriLyt DNA of HCMV, which is critical for transient lytic DNA replication [51]. HMGB1 binds to NP protein of influenza virus in nuclei to enhance the activity of the viral polymerase, which is important for the virus replication [52]. It seems that HMGB1 could specifically bind to either virus encoded proteins or viral genome to influence viral replication cycles. In this study, we found that HMGB1 inhibitor differentially influences virus mRNA expression (Figure 8). Nuclear HMGB1 interacts with nucleosomes, transcription factors, and histones to regulate cellular gene transcription [50]. It will be an interesting study to investigate whether the nuclear HMGB1 could bind to BoHV-1 genome to regulate virus transcription by using CHIP-seq in the future. Especially, it is well known that the virus accomplishes most of the replication cycles in the nucleus, and the virus infection promoted accumulation of HMGB1 protein in the nucleus (Figure 2b and 3a), making it accessible to the de novo virus genome.

Proinflammatory cytokines, such as TNF-α and IL-1β, readily detected in the trachea and lung in BoHV-1-infected cattle, contributes greatly to virus infection-induced rhinotracheitis and pneumonia [53]. We have recently identified that BoHV-1 infection increases the generation of reactive oxidative species (ROS) in cell culture [54], which is also a potential mechanism to mediate the inflammatory response. In this study, we showed that BoHV-1 infection increases the release of HMGB1 (Figure 1d). Of note, the released HMGB1 during the infection of various viruses, such as respiratory syncytial virus (RSV) [16,55], hepatitis C virus (HCV) [56], Herpes simplex virus type 2 (HSV-2) [13,57], and HSV-1 [58], has been convinced to exacerbate severity of the inflammatory disease [12]. For example, the HCV patients with hepatocellular carcinoma (HCC) shows significantly higher levels of serum HMGB1 relative to that of healthy controls (92.1 ± 50.6 ng/mL vs. 7.0 ± 5.9 ng/mL) [59]. Therefore, HMGB1 is accepted as a prognostic biomarker of disease severity due to viral infection [12,60–62]. It is highly possible that HMGB1 is a potential host factor manipulated during BoHV-1 infection to facilitate inflammatory response. Moreover, HMGB1 is a redox-sensitive nuclear protein. The inflammatory mediator ROS can promote HMGB1 release [63,64], HMGB1 is rapidly released after TNF-α stimulation [65], and HMGB1 could enhances TNF-α production [66]. So the produced inflammatory mediators TNF-α, ROS and HMGB1 may establish a reciprocal interaction network with HMGB1 playing a central role in stimulating the inflammatory response during BoHV-1 infection, which is an interesting question to be addressed in vitro, in the future. So our findings will extend our knowledge on the mechanism of the virus infection-induced inflammatory response.

Currently, it is widely recognized that HMGB1, the known chromatin-associated protein, mainly located at the nucleus and maintaining it as an intracellular pool in quiescent cells, and translocation of HMGB1 from nucleus to cytoplasm is a prerequisite for release into the extracellular space [67]. Unexpectedly, our observation in MDBK cells is not follow this dogma because localization of HMGB1 protein in cytosol with high levels and around the rim of nucleus is readily detected in uninfected cells, and cytosol HMGB1 is not readily detected in virus-infected cells (Figure 2a and 2b). Instead, HMGB1 mainly locates at the nucleus following virus infection (Figure 2b). In line with this observation, HMGB1 protein levels were increased in the nuclear fractions but decreased in the cytoplasmic fractions following virus infection as determined by Western blotting (Figure 3a). Thus we suggested that cytosol HMGB1 protein is the source accounting for HMGB1 flowing to different directions induced by virus infection. As such the released HMGB1 protein in the extracellular environment and the increased accumulation of HMGB1 in both nucleus and mitochondria attributed to virus infection mainly came from cytosol. Here, the MDBK cells used in this study were originated from bovine kidney, which are different from what they reported elsewhere. Maybe different species and tissue origins of cells cultures result in this discrepancy. So the manners of HMGB subcellular localization in both quiescent and stimulated (virus infected) MDBK cells are largely different from conventionally accepted, suggesting that HMGB1 functions and manners of flow direction are diversified in different cells.

Interestingly, with confocal microscope we found that HMGB1mainly accumulated in the nucleus and forms typical foci following virus infection, which are rarely observed in uninfected cells (Figure 2a and 2b). Though the essence of these foci has not been elucidated in this study, the typical foci is reminiscent of DNA damage response (DDR) like the canonical γ-H2AX foci which is generally developed in response to DNA damage induced by irradiation because BoHV-1 productive infection in MDBK cells induced DNA damage [68], and HMGB1 plays essential roles in DNA damage repair. For example, HMGB1 binds to DNA damage lesions, bends DNA, thus facilitating recruitment of other DNA damage repair-related proteins to the damage sites induced by psoralen plus UVA irradiation (PUVA) or UVC radiation [7]. While whether HMGB1 is involved in virus infection-induced DDR is currently unknown. If so, part of the accumulated HMGB1 in nucleus may locate at breaks of damaged DNA to facilitate DNA damage repair, therefore the foci are established.

HMGB1 is a critical regulator of mitochondrial function, biogenesis, and morphology [69], favoring repair of damaged mitochondrial DNA [70]. BoHV-1 productive infection induces oxidative stress that contributes to mitochondrial dysfunction [71]. Here, we found that accumulation of HMGB1 in the mitochondria is increased following virus infection (Figure 3b), which is a possible protective response to overcome the injury on mitochondria DNA. Taken together, it is highly possible that the accumulated HMGB1 in both nucleus and mitochondria may facilitate DNA damage repair induced by virus infection, which is an interesting question to be addressed in the future.

By using an experimental type 1 diabetes mouse model, it has been demonstrated that HMGB1 maintains inflammation via modulation of RAGE/AKT1/β-catenin signaling pathway in the diabetic lung [72]. β-catenin/TCF4 heterodimer could directly bind to the promoter regions of HMGB1 genes, thereby leading to transactivation of HMGB1 [44]. Taken these reports together, the signaling between HMGB1 and β-catenin may establish a reciprocal loop to regulate inflammatory response. Our data indicated that BoHV-1 infection leads to depletion of RAGE protein (figure 1f), which may ameliorate the activity of β-catenin stimulated by released HMGB1 via reducing the binding of HMGB1 to RAGE.

iCRT14 could disrupt assemble of β-catenin/TCF complex [45]. HMGB1 protein levels are significantly reduced by iCRT14 in two radioresistant esophageal cancer cells rECA109 and rKyse150, in a context of either with or without ionizing radiation [33]. In line with that report, we found that HMGB1 steady-state protein levels are reduced by iCRT14 in uninfected MDBK cells, while it is not affected in virus-infected cells (Figure 4 A). Interestingly, we found that iCRT14 promotes the release of HMGB1 protein in a context either with or without virus infection (Figure 4c and 4d). And iCRT14 promotes further translocation of HMGB1 into mitochondria following virus infection (Figure 6d). Of note, iCRT14 could significantly reduce virus productive infection [73]. So the observed effects of iCRT14 had on HMGB1 translocation and release were not caused by enhancing virus replication. Taken these data together, we suggested that β-catenin signaling may not only negatively regulate HMGB1 transcription but also affecting the protein translocation and release in virus-infected MDBK cells. Since β-catenin signaling is essential for cell survival, and our data indicated that iCRT14 promotes cell apoptosis induced by the virus infection (Figure 5), which corroborate the increased release of HMGB1 protein. While the mechanism of why β-catenin could negatively regulate HMGB1 subcellular localization and release is currently unknown, which is an interesting question deserving extensive studies in the future.

**Figure 9.**
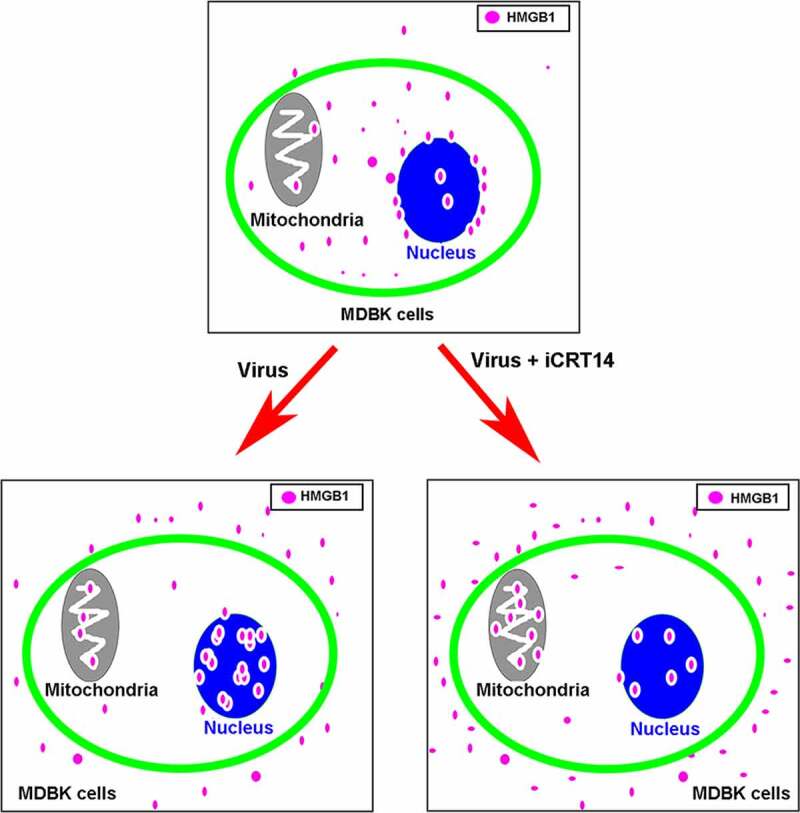
Schematic of putative model on BoHV-1 infection-induced HMGB1 mobilization which is potentially regulated by β-catenin. In the absence of virus infection, HMGB1 distributed in the whole cells, with highlighted staining around the rim of nucleus. Virus infection promotes translocation of HMGB1 into mitochondria and nucleus, as well as release into extracellular spaces. In the presence of iCRT14, HMGB1 release and accumulation in mitochondria induced by virus infection is further enhanced, but the accumulation in nucleus is reduced

Based on our findings we draw a mechanism model as demonstrated in Figure 9 that HMGB1 remains throughout the cells, and concentrates around the rim of nucleus in the uninfected cells. Following virus infection, part of HMGB1 are released into extracellular spaces, while the accumulation in both mitochondria and nucleus is increased, which may culminate in reduced steady-state protein levels of HMGB1. Cytoplasm is the major source of HMGB1 flowing toward different directions. While when β-catenin signaling pathway is inhibited by iCRT14 in virus infected cells, HMGB1 release and accumulation in mitochondria is further increased but it is reduced in nucleus.

In summary, we reported that HMGB1 plays an important role in BoHV-1 productive infection in MDBK cells. And the virus infection leads to alteration of HMGB1 signaling by increasing HMGB1 release, increasing accumulation of HMGB1 protein in both nucleus and mitochondria, as well as relocalization of nuclear HMGB1. Cytoplasm is the major source of HMGB1 flowing toward different directions. Interestingly, for the first time we provide evidence showing that β-catenin signaling may negatively regulate HMGB1 release and mitochondria localization, indicating that β-catenin signaling may regulates inflammatory response partially via effecting HMGB1 release and subcellular localization, which would add on our knowledge on understanding the roles of β-catenin pathway played in virus pathogenesis.

## Data Availability

Data and materials are available upon request.
